# Analysis and Measurement of Barriers to Green Transformation Behavior of Resource Industries

**DOI:** 10.3390/ijerph192113821

**Published:** 2022-10-24

**Authors:** Cunfang Li, Tao Song, Wenfu Wang, Xinyi Gu, Zhan Li, Yongzeng Lai

**Affiliations:** 1 School of Business, Jiangsu Normal University, No. 101 Shanghai Road, Copper Mt. District, Xuzhou 221008, China; 2 Department of Mathematics, Wilfrid Laurier University, Waterloo, ON N2L 3C5, Canada

**Keywords:** resource industries, green transformation behavior, barrier factors, analysis and measurement, Tobit model

## Abstract

To effectively guide and stimulate the green transformation behavior of resource industries and promote the sustainable and high-quality development of the region, it is necessary to deeply analyze and clarify the barrier factors of the green transformation behavior of resource industries. This study measures the green transformation efficiency of the resource industries by selecting the panel data of the mining industry from 29 Chinese provinces, based on the DEA-SBM model, and employing the ideas and methods of system engineering, for the years 2012–2019. Hence, the study employs the Tobit model to verify the factors that hinder the green transformation behavior of the resource industries. The results show that the (1) resource industries’ barriers against the green transformation behavior form a significant barrier effect by inhibiting the efficiency of green transformation; (2) there is a difference in the intensity of the effect of the resource industries’ barriers to the green transformation behavior; (3) regional heterogeneity exists in the effects of the barriers to the green transformation behavior of the resource industries. The findings of the study can provide a scientific basis for further improving the effectiveness of policies related to the green transformation behavior of resource industries.

## 1. Introduction

The resource industry is a collection of enterprises that extract and primarily process exhaustible natural resources, including the coal mining industry, oil and gas extraction industry, and metal and non-metal mineral extraction industry. They have two essential characteristics. First, the significant negative development externalities. Resource industries’ mining behavior will produce severe environmental damage and ecological declines such as the water system destruction, surface subsidence, land sanding, and atmospheric pollution in the mining areas, thus threatening the sustainable development of the regional economy and society. Second, they have a significant lifecycle. Since the development of resource industries and resource reserves have an inverse coupling relationship, resource industries show the life cycle of “initial production–increased production–stable production–declining production”. According to the data regarding mining in China, l67,000 mines and more than 3.75 million hectares of land have been damaged, collapsed, or occupied [1,2]. About 795 million tons of gangue and 6004 million m^3^ of mine water are being discharged annually, thus damaging about 2.2 billion m^3^ of groundwater resources [3]. In Yulin City, Shanxi Province, of the original 115 springs in the Zhangjiamao area, 102 springs have been depleted after the mineral development, and the total flow has been reduced by 95.8%. In the northern part of Shenmu County, there are 869 original lakes and 79 lakes have been left dry after mineral development [4]. The negative impact of mining on the environment reaches 65–75% [5]. Further investigation shows that for the non-ferrous metal industry, 355 mines have been either closed or will be closed, thus accounting for 46% of the total number of mines. For the coal industry, among nearly 600 mines in the original 94 key enterprises, about one-third of the mines have entered the resource depletion stage and more than 120 mines face closure. Among the 2000 plus local enterprises, the percentage of resource depletion mines is even higher [6]. Resource depletion undoubtedly poses a direct threat to the sustainable development of the resource industry itself and is highly likely to lead to social stability problems [7,8,9,10,11].

The achievement of sustainable development by the resource industries under the dual challenges of environmental constraints and resource depletion is a global issue. Certain experiences from abroad are difficult to emulate owing to China’s significant differences between the resource management and the enterprise organization methods. The development of the resource industries in China are also accompanied by certain unique issues such as the construction and challenges of the new development pattern, the transformation and revitalization of resource-based cities, the soundness and improvement of the social security system, and the construction and sublimation of a beautiful China. These unique issues make the situation particularly complex. Concomitantly, the green development has become a new driving force for the economic development and environmental governance. The 18th Party Congress has proposed to vigorously promote the construction of ecological civilization and reverse the trend of ecological environment deterioration. The 19th Party Congress has proposed to establish a sound economic system of green, low-carbon, and cyclic development, thus providing new ideas for the development of the resource industries. This is to crack the problem of sustainable development. Therefore, the green transformation behavior has become an inevitable choice.

The green transformation behavior of resource industries refers to the upgrading of the resource industries’ position in the industrial chain and value chain through the conversion between different industries or development modes. This is to form a sustainable and high-quality development mode with high efficiency, low consumption, less emission, and cleanliness. The essence of such behavior is the green transformation of the production process, green reconstruction of operation system, and green transformation of economic growth mode of the enterprises in the industry. In the face of such significant changes, Chinese resource industries should firmly grasp the development opportunities and make every effort to implement green transformation. However, an in-depth investigation of the green transformation practices of the Chinese resource industries have found that the different enterprises always have different paces and even “beggar-thy-neighbor” transferring of pollution in the industry [12]. This indicates that the implementation of the green transformation behavior of resource industries has encountered enormous barriers. There are questions regarding the barriers for implementing the green transformation behaviors in the resource industries, and the degree of the constraint of these barriers. Further, we need to know about the consistency of the barriers among the resource industries in different regions. The realization of the objective of systematic investigation of the above scientific questions is not only conducive to the resource industries to accurately identify and break through the possible barriers in the process of green transformation and steadily promote the green transformation practice efficiently, but also can promote the relevant governments to improve the effectiveness of the incentive policies for the green transformation of resource industries and the sustainable and high-quality development of the region, and can broaden the knowledge of the scholars on the theory of organizational behavior such as the green transformation of resource industries.

## 2. Literature Review

The green transformation behavior, owing to its importance for the development of the resource industries, has been intensely researched. According to the relevant literature, the current domestic and international research primarily focuses on the following three aspects.

First, the path issue of the green transformation behavior of resource industries is considered. Abuzeinab et al. (2016) [13] concluded that the business model reshaped from the five aspects, viz., green value proposition, target group, key activities, essential resources, and financial logic, is the core of the green transformation behavior of the resource industries. This argument suggests an essential path of the green transformation behavior of the resource industries. A study on the resource industries in Australia has found that the proactive and preventive management of mine waste provides significant environmental benefits and gains. This can be considered as a contribution of the resource industries to circular economy goals as long as minerals can be extracted at an acceptable environmental cost, while the loss of non-renewable resources can be minimized [14]. This argument also indicates the path of emission reduction for the green transformation behavior of the resource industries. Other scholars divide the transformation stages of the resource-based enterprises based on the enterprise lifecycle, and argue for the feasibility of the path selection of the green transformation behaviors carried out by the enterprises in different transformation stages through multiple cases [15]. This argument also reflects the systematic thinking on the path selection of the green transformation behavior.

Second, the issue of evaluating the green transformation behavior of resource industries is considered. Gorman and Dzombak (2018) [16] concluded that the evaluation strategy for the sustainability of the resource industries’ mining operations includes the measuring, monitoring, and attempts to improve various environmental performance indicators. However, the critical indicators of the resource industries’ environmental performance involve the resource consumption efficiency, minimizing land destruction, reducing pollution, and reclamation of depleted mine sites. Owing to the heterogeneity of the environmental indicators reported by different resource-based enterprises, comparison of the environmental performance among them is challenging. Therefore, certain scholars have proposed a comprehensive evaluation framework that identifies nine major environmental indicators based on the international sustainability guidelines. They have evaluated the environmental performance data of 11 top global oil and gas companies, and ranked them for a comparison [17]. Certain scholars have analyzed the economic relationship between the green transformation behavior of resource industries and spatial development, and established a quantitative assessment model of the intersectoral relationships and green transformation of the resource industries within the economic framework of the resource-based cities. This analysis focuses on assessing the dominance of the resource industries in the transformation of the specific regions, besides their role in the economic development of resource-based cities [18]. Other scholars have constructed an evaluation index system from multiple dimensions, and used the fuzzy set comparison analysis and entropy weight methods to comprehensively evaluate the green transformation behavior of the resource-based enterprises [19,20]. Further, they have concluded that the green transformation behavior of the resource-based enterprises in China remains in the transformation stage of “innovative but not green,”. Hence, resource-based enterprises need to increase the green technology continuously. It is necessary to continuously increase the green technology investment, improve incentive policies, and strengthen the supervision to promote green transformation and sustainable development. Obviously, these assessments reflect the degree of green transformation and performance of the resource industries to a certain extent.

Third, the issue of factors influencing the green transformation behavior of resource industries is considered. Kesidou and Demirel (2012) [21] concluded that strict environmental regulation policies are the driving force for the green innovation in resource industries based on a survey and empirical analysis of 1566 enterprises in the United Kingdom. Further studies have concluded that the environmental regulations negatively and positively impact the enterprises with low and high production efficiency, respectively. Other scholars have used the data on heavily polluting industries such as coal and steel to demonstrate that the green tax incentives promote the green transformation of industries, though there are heterogeneous differences in the regional property rights structures and degrees of marketization [22,23]. Certain scholars, based on industry-level studies, have also concluded that the optimization of the resource industry structure could significantly promote the transformation behavior of the resource-based enterprises [24]. However, studies based on the enterprise levels have concluded that the heterogeneous characteristics of the top management team of enterprises could influence the formulation, adjustment, and change of enterprise strategies, which in turn affects the performance of the green transformation behavior of enterprises [25]. Furthermore, certain scholars have studied the influencing factors of the green transformation of industrial enterprises to the barrier factor level in depth. For example, the group from the Institute of Industrial Economics, Chinese Academy of Social Sciences (2011) [26] pointed out the imbalance of costs and benefits as the internal barrier of green transformation of enterprises. Accordingly, the external barriers to green transformation are the unreasonable assessment and promotion mechanisms of the local government officials, incomplete reform of factor price formation mechanism, the considerable risk of technological innovation, application and transfer, unsound environmental compensation mechanism, and imperfect social supervision system.

The green transformation behavior of resource industries has been studied from various perspectives, and henceforth, achieved fruitful results. However, there are still certain shortcomings in the existing studies, primarily in the emphasis on the excavation of the influencing factors of the green transformation behavior of resource industries without focusing on the barrier factors. Certain studies simply state the primary role of the relevant barrier factors without the systematic analysis and accurate classification of the barrier factors, besides the scientific measurement and in-depth comparison of the constraint strength of the barrier factors [27]. The lack of a theoretical investigation for such issues makes the scientific guidance for the implementation of green transformation behaviors of the resource industries insufficient. Thus, it is difficult to improve the efficiency of green transformation of the resource industries effectively and efficiently. These issues are the central theme for this paper.

According to the characteristics and environment of the resource industries, this paper comprehensively combs through the barrier factors of the resource industries’ green transformation behavior through the interviews with theexperts and GEM (groundings, enterprises, markets). Further, we analyze the action mechanism of the barrier factors, scientifically measure the efficiency of resource industries’ green transformation, and probe into and demonstrate the extent of the restriction.

The rest of this paper is structured as follows. In Section 3, we systematically analyze the barriers to the green transformation behavior of resource industries and their mechanisms of action based on the interview survey and GEM model. In Section 4, we conduct the variable design and measurement of the implementation efficiency of the green transformation behavior of resource industries and their barriers. In Section 5, we conduct the empirical tests and analysis of the barriers to the green transformation behavior of the resource industries. In Section 6, we give our conclusions and the ensuing policy implications. In Section 7, we point out the limitations of the article and present the research outlook.

## 3. Systematic Analysis of the Factors That Hinder the Behavior of Resource Industries in Green Transformation

**Figure 1:**
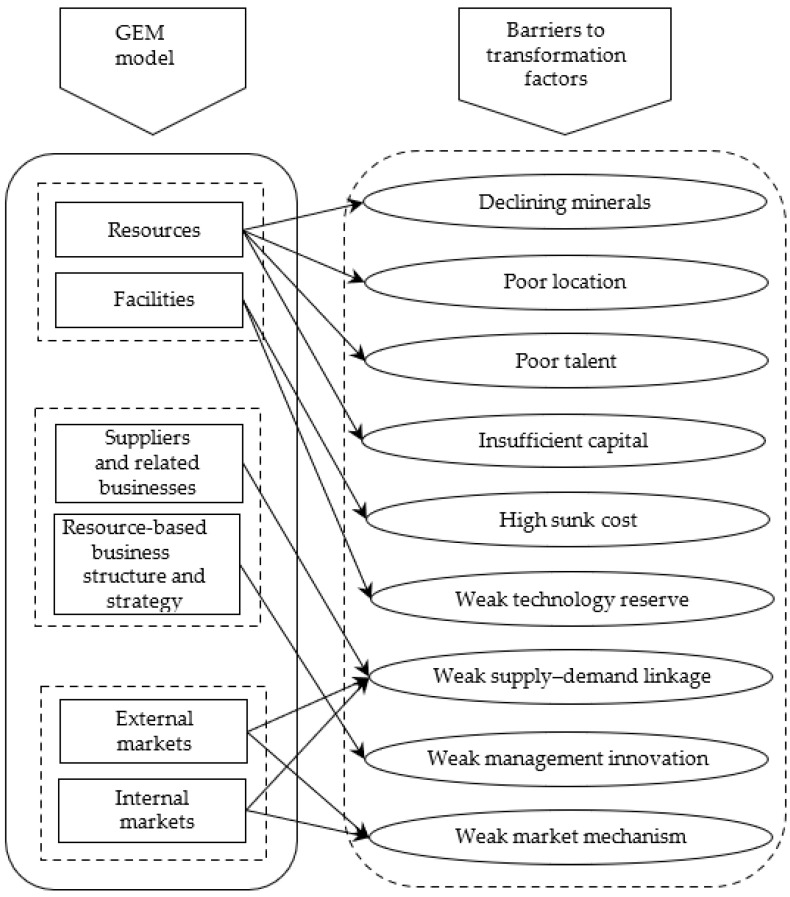
GEM analysis model based on resource industries’ green transformation behavioral barriers convergence chart.

Since the concept of “green economy” was introduced by the British environmental economist Pierce in his book, “Blueprint for a Green Economy” in 1989, the green transformation has evolved in to a critical path for a green economy. It has become the focus of discussion among academics, governments, and industries. As an essential part of the green transformation, the green transformation behavior of resource industries is sophisticated since they need to overcome the pollution impact of the general industries on the environment, and mitigate the direct damage to the environment caused by their resource development [28]. Compared with the traditional “black” development model of the resource industries, the connotation of the green transformation behavior is dynamic and encompasses all aspects of the entire development process’s supply chain and value chain. The green transformation behavior faces four conditions, viz., the dynamic changes of operational boundary, constant expansion of the scope of environmental damage, the gradual increase in the degree of pollution, and limitations of the regional resources and environmental carrying. Further, it copies with the gradual improvement in human awareness of the environmental impact of resource development, particularly with the commitment of China’s “double carbon” target contribution [29]. The mission is ambitious, whereas the task is heavier. Concomitantly, the green transformation behavior depends on the innovation of the operation concept, development of technology, and management system, besides the support of talents and financial investment. Significantly, the innovation of the green development technology is the green upgrade and transformation of the traditional development technology, besides the active development of the novel technology, material, way of recycling, and ecological restoration. Thus, the investment is more, and the risk is higher. For implementing such a complex system project, government promotion, market compulsion, and public support are needed, besides the active participation of enterprises [30,31]. Since the practice of the green transformation at the level of resource industries in China has been limited and the effect of the practice varies significantly, it is necessary to systematically analyze the problems associated with the evolution of green transformation behaviors of resource industries, and hence to eliminate the barriers to the green transformation behaviors of resource industries. This is an important prerequisite for promoting the green transformation practices of resource industries. Accordingly, the persons in charge of the large enterprises promoting the construction of green mines, such as Xuzhou Mining Group in Jiangsu Province, Shandong Energy Group, and Hebei Jizhong Energy Group, have been selected for conducting semi-structured interviews regarding the barrier factors of the green transformation behaviors of resource industries. Certain practice perception factors have been initially obtained. Then the characteristics of the resource industries and the connotation of the green transformation in China have been analyzed in depth. Referring to the results from the existing literature [32] and drawing on the systemic factor analysis model, GEM [33,34], which was improved by Tim and Henrev in 1998, we analyze three “factor pairs”, i.e., six factors, to reveal the barriers to the green transformation behavior of resource industries, as shown in Figure 1.

The first “factor pair” is the groundings, which is the supply factor of the green transition system, including both the resources and facility factors.

(1) Reviewing the aspect of resources.

Traditional resource industries rely on the exploitation of the mineral resources and primary processing methods to obtain a rapid development. Green transformation behavior is neither a complete negation of the traditional development methods, nor a complete exclusion of the mineral resources, or relying on the mineral resources without depending on mineral resources, and jumping out of them without giving up mineral resources. Hence, regardless of the implementation of this transformation between different development models or different industries, mineral resource endowment still has an important supporting role for sustainable industrial development. This non-renewable and potentially depleting mineral resource endowment will weaken the supply of the green transformation system and hinder the implementation of the green transformation behavior to a certain extent, and decay as the high-intensity exploitation. Concomitantly, the green transformation behavior of the resource industries relies on the drive of the technological and management innovation. Such innovation requires both the talent and financial input. Owing to the specificity of the mineral resource endowment area, the resource industries are often remotely located from the economically developed areas, with closed transportation and a monotonous culture. The operating income is subject to the policy regulation and control, and hence it is difficult to lure high-level technical and management talents. When the resource endowment gradually declines, excellent talents are easily lost. Since innovation is highly uncertain and risky with additional capital investment and an extended recovery period, the input cost of the resource industries cannot be compensated quickly, which is likely to cause embarrassment to the current development. Most enterprises in industries do not even have sufficient funds to ensure the smooth operation of a green transformation system in this unchartered field. Thus, the green transformation behavior is often challenging. As a result of the above theoretical analysis, the following hypothesis can be formulated.

**Hypothesis** **1a** **(H1a).** *Declining minerals have a hindering effect on the green transformation behavior of resource industries*.

**Hypothesis** **1b** **(H1b).** *Poor location has a hindering effect on the green transformation behavior of resource industries*.

**Hypothesis** **1c** **(H1c).** *Poor talent has a hindering effect on the green transformation behavior of resource industries*.

**Hypothesis** **1d** **(H1d).** *Insufficient capital has a hindering effect on the green transformation behavior of resource industries*.

(2) Reviewing the aspect of facilities.

Resource industries, as a class of entities engaged in developing the mineral resources and capital-intensive enterprises, require a considerable early investment in the special equipment and facilities to explore, extract, and process the mineral resources. Along with the green transformation, the original sloppy development method is gradually abandoned and replaced by the green production methods, which will incur high sunk costs for the green transformation owing to the strong asset specialization and high fixed costs. Like other general industries, gaining profits is the essence of the survival and development of the resource industries. The fear of significant sunk cost losses will undoubtedly weaken the supply of the green transformation systems and hinder the pace of the green transformation behavior. Furthermore, resource industries thrive on mining. With the mature technologies in surveying, mining, and primary processing of resources, they provide essential resource goods for the social and economic development and people’s livelihood, and achieve a rapid development through the daily repetitive (and rough) production and operation activities. Thus, they also lack the pressure and motivation for technological innovation and environmental protection, besides a lack of awareness of the technological innovation and environmental protection upgrading. Their technological reserves are relatively weak [35,36]. Owing to the depletion of mineral resources, it is difficult for the enterprises to continue their traditional production methods and rely on their technological reserves to promote green transformation. As a result of the above theoretical analysis, the following hypothesis can be formulated.

**Hypothesis** **1e** **(H1e).** *A high sunk cost has a hindering effect on the green transformation behavior of resource industries*.

**Hypothesis** **1f** **(H1f).** *A weak technology reserve has a hindering effect on the green transformation behavior of resource industries*.

The second “factor pair” is the enterprises, which are the structural factors of the green transformation system, including the outer layer of suppliers and related enterprises, and the inner layer of resource-based enterprises’ structure and strategy. The suppliers and related enterprises represent the goods and services of other enterprises related to the supply and demand of the resource industries. The structure and strategy of the resource-based enterprises represent the management innovation related to the strategic direction and competitive strategy of the resource-based enterprises.

(1) Reviewing the aspect of the outer layer suppliers and related enterprises.

In the operation of the resource industries, the resource industry chain is formed by undertaking the transfer of technical service materials and supply of various development and processing equipment from upstream resource exploration units on the one hand, and exporting resource products or raw materials to downstream industrial enterprises and consumers through its own organizational development and processing on the other. The resource industry chain is formed by the linkage of the enterprises in different exploration, exploitation, and processing stages, with the resource-based enterprises as the leading core. The enterprises in the chain form a closed and symbiotic structure of industries through the synergy of the division of labor, supply, and demand linkage, technical cooperation, and information transfer, which in turn promote the effective connection of each link in the chain [37,38,39]. Changes in the endowment of the mineral resources and technological upgrades in development and processing equipment will be transmitted through the upstream supply relationship of the industry chain. Similarly, the upgrading of market demand for resource products will also form feedback through the downstream demand relationship of the industry chain, forcing or stimulating resource industries to implement green transformation behaviors. Once the linkage between the supply and demand is weakened, the information transmission and operational efficiency of the whole industrial chain will be significantly reduced, which will inevitably weaken the kinetic energy of the resource industries’ green transformation behaviors. As a result of the above theoretical analysis, the following hypothesis can be formulated.

**Hypothesis** **2a** **(H2a).** *A weak supply–demand linkage has a hindering effect on the green transformation behavior of resource industries*.

(2) Reviewing the structural and strategic aspects of resource-based enterprises from the inner layer.

The green transformation behavior of resource industries is essentially a structural and strategic transformation, that is, the green transformation of the production process, the green reconstruction of the operation system, and the green transformation of the growth mode of the enterprises in the industry. To achieve such a significant change, the key lies in the innovation of the management concept of decision makers and the innovation of the management behavior of executors, so that new management elements (such as new management methods, new management tools, and new management models) or combinations of elements can be effectively introduced into the management system of each enterprise to further strengthen the kinetic energy of the green transformation behavior of resource industries [40]. However, owing to the remoteness of the resource endowment, the majority of the resource-based enterprises are distributed in the regions that are lagging in development, having inconvenient transportation, and a lack of talents. For an extended period, the enterprises run the society in a relatively closed development. The enterprise management follows the traditional concepts and ideas that are relatively backward. The organizational structure is more redundant and rigid, thus making it difficult to absorb new management methods, means, and models, and hence difficult to form practical management innovation. The pace of the green transformation behavior of resource industries is hindered to a certain extent. As a result of the above theoretical analysis, the following hypothesis can be formulated.

**Hypothesis** **2b** **(H2b).** *A weak management innovation has a hindering effect on the green transformation behavior of resource industries*.

The third “factor pair” is markets, which are the demand factors of the green transition system, including both the external and internal markets. The external and internal markets indicate the international and domestic markets, respectively. The market is an essential means of the resource allocation, and its recognition of green products can guide the development direction and green transition behavior of resource industries [41]. Alternatively, the economic kinetic energy that promotes the green transformation behavior of the resource industries arises from the marginal and risk incentives under the operation of the market mechanism. Among them, the marginal incentive refers to the fact that, owing to the continuous evolution of the resource industry chain, the comparison of business returns between different industries or development stages of the resource industries, before and after the emergence of green transformation behavior, morphs continuously. Such differences and changes alter the marginal cost and marginal revenue of the production through the market competition mechanism and change the marginal profit so that the input scale of the original production before the transformation is suppressed. Concomitantly, the input scale of the new products after the transformation is increased. Risk incentive refers to the business risks and the corresponding business returns between different industries or development stages, before and after the green transformation behavior of resource industries, which constantly evolve under the market competition mechanism, thus forming the risk–return differences. This difference causes each enterprise to compress the input scale of the original production before the transformation, and increase the input scale of the new product after the transformation, according to their different risk preferences, risk tolerance, the risk–return reciprocity principle, and risk preference screening mechanism. The result of these two incentives ensues a change in the ratio of the old and new production structures to promote green transformation. The effectiveness of these two incentives depends on the strength of the market mechanism. Therefore, when the market mechanism is robust, the market is competitive and the marginal profit is low. The marginal incentive and risk incentive of the green transformation behavior of the resource industries are enhanced, which can promote the green transformation behavior. Otherwise, it is not. Owing to the current reversal of the course of economic globalization, the international market entry barriers are complicated and coupled with the impact of the COVID-19 epidemic. Global supply chains and industrial chains have been partially broken, and the downward pressure of recession has increased significantly. The domestic market mechanism is gradually improving, though the environmental cost accounting of the resource industries is not in place. Non-market pricing still exists. The prices of the resource products are distorted. The market response is not accurate enough. The leverage is not significant enough. As a result of the above theoretical analysis, the following hypothesis can be formulated.

**Hypothesis** **3** **(H3).** *A weak market mechanism has a hindering effect on the green transformation behavior of resource industries*.

## 4. Quantitative Measurement of the Barriers to Green Transformation Behavior of Resource Industries

### 4.1. Model Setting

To test the degree of constraints of the above preliminarily obtained barriers on the green transformation behavior of resource industries, the paper used the variable that can effectively measure the effect of implementing the green transformation behavior on resource industries with green transformation efficiency as the explanatory variable. An econometric analysis was conducted using the preliminary obtained barrier factors as explanatory variables. In addition, considering that the range for the effective value of green transition efficiency is greater than 0, which is truncated data and meets the conditions of Tobit model applicability, the following benchmark model was constructed, where *Y* is the general explanatory variable, *X* is the general explanatory variable, *i* is time, *t* is individual, and *α* is the constant term.

(1) $$Y_{it}=\{\begin{array}{c} \alpha_{0}+\beta_{i}X_{it}+\varepsilon_{it},Y_{it}>0\\ 0,Y_{it}\leq 0 \end{array}$$

Refine the benchmark model as follows.

(2) $$G_{it}=C+\beta_{1}res_{it}+\beta_{2}tr_{it}+\beta_{3}edu_{it}+\beta_{4}alr_{it}+\beta_{5}dep_{it}+\beta_{6}cap_{it}+\beta_{7}cn_{it}+\beta_{8}mc_{it}+\beta_{9}mi_{it}+\varepsilon_{it}$$

*G* is the green transformation efficiency of the resource industries, and *res* is the mineral endowment. Further, *tr* is the location status and *edu* is the talent level, whereas *alr* is capital stock and *dep* is sunk cost. Moreover, *cap* is technology stock, *cn* is the supply and demand linkage, *mc is* the management innovation capability, *mi* is the market mechanism effectiveness, *i* is the province and *t* is period, *β* is a coefficient to be determined, *C* is a constant term, and *ε* is a random error term.

### 4.2. Measurement of Variables

We selected the mining industry panel data from 29 Chinese provinces, during 2012–2019 (Tibet and Hong Kong, Macao, and Taiwan are missing from the specific data, and Shanghai was excluded owing to the discrepancy between the data from Shanghai and various other provinces), to empirically test the barriers to the green transformation behavior of the resource industries. Specific data were obtained from the China Industrial Statistical Yearbook, China Taxation Yearbook, China Science and Technology Statistical Yearbook, China Environmental Statistical Yearbook, China Sub-Provincial Marketization Index Report, and China Statistical Yearbook, besides the statistical yearbooks and statistical bulletins of each province. The data of the monetary category indicators were deflated using 2012 as the base period. Data of green invention patent applications were obtained from the CNRDS database. The SPSS-based serial mean method was employed to complete the missing data.

#### 4.2.1. Green Transformation Efficiency Measurement

In measuring the efficiency of the green transition, not only the inputs of multiple factors of production should be considered, but also multiple outputs should be included in the decision-making system. In particular, the examination of non-desired outputs can better reflect the effect of implementing the green transition. The SBM model with non-expected outputs proposed by Tone not only integrates the multiple-input and multiple-output problem, but also solves the slack problem of traditional DEA models that do not consider inputs and outputs [42], avoiding the bias caused by radial and angular selection. Hence, considering that the returns to scale of different extraction technologies in resource industries are at different stages, this study measured the efficiency of green transformation of resource industries based on the non-expected output SBM model in the case of variable returns to scale [43]. The model is constructed as follows.

It was assumed that the quantity of decision making units (DUM) is *n*, each having 3 variables as input, desired output, and non-desired output, symbolically denoted as *x∈R^q^*, *y^g^∈ R^u^_1_*, *y^b^∈ R^u^_2_*, and the matrices *X*, *Y^g^*, *Y^b^* can be defined.

(3) $$\begin{array}{c} X=\left[x_{1},\cdots ,x_{n}\right]\in R^{q\times n}>0\\ Y^{g}=\left[y_{1}^{g},\cdots ,y_{n}^{g}\right]\in R^{u_{1}\times n}>0\\ Y^{b}=\left[y_{1}^{b},\cdots ,y_{n}^{b}\right]\in R^{u_{2}\times n}>0 \end{array}$$

The set of production possibilities *P* containing non-desired outputs was constructed as follows.

(4) $$P=\{\left(x,y^{g},y^{b}\right)|x⩾X\lambda ,y^{g}⩽Y^{g}\lambda ,y^{b}⩾Y^{b}\lambda ,\lambda ⩾0\}$$

In the case of variable payoffs of scale, the SBM model considering undesired output has the following form.

(5) $$\rho =\min\frac{1-\frac{1}{q}\sum_{i=1}^{q}\frac{s_{i}^{-}}{x_{i0}}}{1+\frac{1}{u_{1}+u_{2}}\left(\sum_{r=1}^{u_{1}}\frac{s_{r}^{g}}{y_{r0}^{g}}+\sum_{i=1}^{u_{2}}\frac{s_{l}^{b}}{y_{l0}^{b}}\right)},\;s.t\left\{ \begin{array}{l} x_{0}=X\lambda +s^{-}\\ y_{0}^{g}=Y^{g}\lambda -s^{g}\\ y_{0}^{b}=Y^{b}\lambda +s^{b}\\ \sum_{i=1}^{n}\lambda_{i}=1,s^{-}⩾0,s^{g}⩾0,s^{b}⩾0 \end{array}\right.$$

In Equation (5), *x, y^g^, y^b^* represent the input, desired output, and undesired output variables of the decision unit separately. *s^−^*, *s^g^*, *s^b^* represent the input, desired output, and undesired output slack variables separately. *λ* is the weight vector and the subscript “0” is the evaluated unit. *ρ* is the efficiency value, which takes a value between 0 and 1.

The essence of green transformation of resource industries is to achieve a win–win situation for economic, ecological, and environmental benefits. Since ecological and environmental benefits are externally uniform, they were considered as a whole in the research framework. As a result, in order to measure the efficiency of green transformation of resource industries comprehensively and accurately, the environmental efficiency considering non-desired output and the economic efficiency excluding non-desired output were incorporated into the green transformation efficiency measurement system, and the specific measurement model was constructed as follows.

(6) $$G_{it}=S_{it}/E_{it}$$

In Equation (6), *G_it_* is the green transformation efficiency of the resource industries and it represents the environmental efficiency corresponding to one unit of economic efficiency. *S_it_* and *E_it_* represents the environmental efficiency and economic efficiency, respectively, of the resource industries. The specific input–output indicators were selected as follows:

(1) Input indicators. Input indicators include the capital, labor, and energy inputs. Among them, the capital input was measured by the year-end net fixed assets of the mining industry in each province, and labor input was measured by the year-end average number of the employees in the mining industry in each province, whereas the energy input is measured by the year-end total energy consumption in the mining industry in each province [42].
(2) Output indicators. Output indicators include the desired output and non-desired output indicators. Drawing on the study of Hu and Ding (2020) [44], the primary business income of the mining industry in each province was used as a measure of the desired output. The resource industries inevitably bring in the emissions of sulfur dioxide, wastewater, and solid waste owing to the negative environmental externalities derived from their production and operation processes. Therefore, sulfur dioxide emissions, wastewater emissions, and solid waste generation were selected and integrated into a comprehensive environmental pollution index by employing the entropy value method [45] as a measure of the non-desired output.

**Figure 2:**
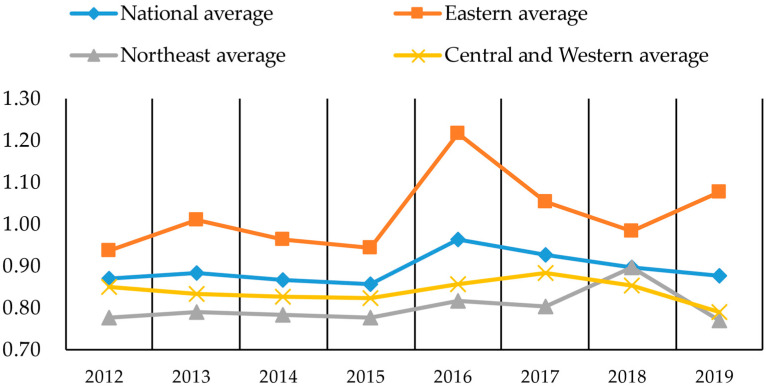
Trend of green transformation efficiency of resource industries in three major regions of China, 2012–2019.

The above index data were brought into MATLAB to derive the green transformation efficiency of the resource industries, and the results are shown in Table 1. Further, the changing trend of the green transformation efficiency of the resource industries is plotted in three major regions, as shown in Figure 2.

According to Figure 2, the overall green transformation efficiency of the resource industries in China shows a fluctuating upward trend, though the growth is minimal. The green transformation efficiency as of 2019 is only 0.01 higher than that of 2012, which indicates that the green transformation process of the resource industries in China had been prolonged. The green transformation efficiency of the resource industries in the eastern region is much higher than that of other regions, followed by the central and western regions, and the lowest in the northeast. The green transformation behavior of the resource industries in China seems to encounter more resistance. The green transformation behavior in different regions has different paces, which also confirms the previous realistic observation and theoretical analysis of the green transformation behavior of the resource industries, given in this paper. Nevertheless, it is urgent and necessary to analyze the barriers to the green transformation behavior of the resource industries and the extent of their constraints in depth.

#### 4.2.2. Barrier Factor Measurement

For the resource industries, the variables of the behavioral barriers to the green transformation were designed as shown in Table 2. Descriptive statistics were compiled and grouped with respect to the regions using the relevant databases and Stata 16.0. The results are shown in Table 3.

(1) Mineral endowment (res). The fixed asset investment is found to be positively correlated with the scale of the mineral endowment, while considering that the resource industries develop more varieties and forms of the mineral resources. We used the proportion of the fixed asset investment in the mining industry in each province to the total social fixed asset investment, for unifying the scale of the mineral endowment regarding the study of Meng and Zhang (2020) [46].
(2) Location status (tr). Since the taxation is an administrative measure to regulate the enterprise behavior, the taxation revenue can reflect the change of the government attitude toward the green transformation behavior of the resource industries. Thus, the regional taxation revenue was used as an indicator to measure the location status. When the annual taxation revenue decreases or increases, the local location policy advantage increases or decreases, respectively.
(3) Talent level (edu). Referring to the study of Zhu Dongbo and Ren Li (2017) [47], each province’s average number of years of the personnel education was utilized to measure the talent status. There are more opportunities such that the resource industries can absorb talent from the local area, if the average number of years of education of the local personnel is longer. Accordingly, the higher the level of talent, the weaker the talent constraint of enterprises’ green transformation behavior is weaker for a higher level of talent. The formula can be given as, average years of education = (number of illiterate people × 1 + number of people with elementary school education × 6 + number of people with junior high school education × 9 + number of people with high school and secondary school education × 12 + number of people with college and bachelor’s degree or above × 16)/total population over six years old.
(4) Capital stock (alr). The ratio of liabilities to the current assets reflects the ability of the enterprises to finance their operations. Financing is one of the main methods for each enterprise to expand their production scale, develop markets, and obtain high profits and transformation funds. Therefore, the ratio of liabilities to current assets in the mining industry in each province was used to measure the capital stock status. The enterprise’s financing ability is weaker for a higher ratio. The green transformation behavior of the resource industries is stronger for a corresponding capital stock that is scarce.
(5) Sunk cost (dep). The sunk cost is a non-transformation input cost that inevitably exists when the green transformation behavior of the resource industries has been generated. Cumulative depreciation was used to measure the sunk costs. The fixed assets and equipment of the resource industries is more for a higher accumulated depreciation. The sunk cost will be higher in the face of green transformation owing to its strong specialization, and the constraint on the green transformation behavior of the resource industries will be stronger.
(6) Technology reserve (cap). Green invention patents are more green-oriented and technologically innovative than other types of patents, bringing substantial impact to green transformation [48,49]. Meanwhile, a patent application is an act of seeking legal protection for a technological innovation that has already been achieved. Hence, the number of green invention patent applications was used to measure the technology reserve status, i.e., the greener the invention patent applications, the stronger the technology reserve.
(7) Supply and demand linkage (cn). Owing to the increasing number of registered legal persons in upstream and downstream of the resource industries, the supply and demand are expanding. Enterprises in industries prefer more profitable cooperation when they encounter more opportunities to choose from. To a certain extent, it weakens the stability of the supply–demand relationship of the industry chain. Therefore, the number of registered legal persons upstream and downstream of the resource industries in each province wasused to measure the strength of the supply–demand association. The constraint for the green transformation of resource industries is stronger for a weaker supply–demand linkage of the industrial chain.
(8) Management innovation capability (mc). According to the study of Chun and Tingting (2019) [50], the comprehensive index for the management innovation was used to measure the management innovation capability. The index system has been constructed by selecting the management expense, sales expense, management expense contribution, inventory turnover, current asset turnover, total asset turnover, and sales expense contribution of the mining industry in each province. We used SPSS to conduct principal component analysis. The KMO statistics exceeds 0.5. Several principal components were integrated into a comprehensive index of the management innovation, which was employed to measure the strength of the management innovation ability of the resource industries.
(9) Market mechanism validity (mi). The marketization index starts from six aspects: (1) the relationship between government and market, (2) the development status of the non-state economy, (3) product market development, (4) factor market development, (5) intermediary market organization, and (6) legal system improvement. It adopts the objective indicators that can better reflect the depth and breadth of the marketization reform in each region. The marketization index of each province was used to measure the validity of the market mechanism of the green transformation behavior of the resource industries, according to Yu et al. (2010). This is to complement the calculation of the marketization index of the sample year [51].

As can be seen from Table 3, the descriptive statistical analysis of the grouped variables after standardization shows that the barrier factor variables have regional differences. Combined with the differences in green transformation efficiency in Figure 2 and Table 1, we can initially determine that the above-mentioned barrier factors have an impact on the green transformation efficiency of resource-based enterprises. What kind of impact do these barriers have on the green transformation efficiency of resource-based industries nationwide? What is the degree of impact? What are the impact paths?

## 5. Empirical Analysis of Factors That Impede the Behavior of Resource Industries in Green Transformation

### 5.1. Basic Regression Analysis

By testing all the barrier factor variables for multicollinearity, the results demonstrate that the variance inflation factor (VIF) is less than 10, which indicates that there is no multicollinearity in the variables. Based on the above bilateral Tobit model, Stata 16.0 was employed to test the degree of constraints for each barrier factor variable on the green transformation behavior of the resource industries. The results are listed in Table 4.

According to Table 4, the talent level term and the management innovation capability term are significantly positive at the 1% and 5% levels, respectively, indicating that the higher the local resource industry measure, the higher the efficiency of green transformation, and vice versa. Proving that poor talent and weak management innovation will hinder the green transformation behavior of resource industries, H1c and H2b are confirmed. In addition, the capital stock term, the sunk cost term, and the supply and demand correlation term are all significantly negative at the 1% level, indicating that the higher the local resource industry measure, the less efficient the green transition is, and vice versa. It is proven that insufficient capital, high sunk cost, and weak supply–demand linkage are the obstructive factors for the green transformation behavior of resource industries, and H1d, H1e, and H2a are confirmed. Meanwhile, Table 4 also shows that the coefficient of the mineral endowment term is significantly negative at the 5% level, indicating that the higher the mineral endowment measure of the local resource industry, the lower the efficiency of green transformation. In essence, it reflects that the current resource-based enterprises in China are still highly committed to mineral resource development and primary processing. The rich mineral endowment has a “crowding-out effect” on green technology innovation, which makes resource industries rely too much on resource advantages and weakens transformation momentum, forming barriers to transformation. The coefficient of the location status term is significantly positive at the 1% level, indicating that the higher the local location status measure, the higher the efficiency of green transformation of resource industries. In essence, it reflects that the higher the tax revenue of local government and the worse the location policy advantage, the higher the efficiency of green transformation of resource industries, which further indicates that the poor location “pushes” the green transformation behavior of resource industries. The coefficient of the technology reserve term is insignificant, indicating that the current resource industries mainly rely on policy-driven passive transformation. In addition, because of the high uncertainty and risk of green technology innovation, the technology reserve tends to maintain the traditional production method, then the impact of technology reserve on the efficiency of green transformation is also uncertain. The coefficient of the market mechanism validity term is significantly negative at the 1% level, indicating that the higher the local market mechanism validity measure indicator, the lower the efficiency of green transformation of resource industries. In essence, it reflects that the higher the level of local marketization, the fiercer the market competition, the lower the marginal profit, the stronger the marginal incentive and risk incentive for green transformation behavior of resource industries should be, yet the green transformation behavior is hindered. All these further indicate that the declining minerals, poor location, weak technology reserve, and weak market mechanism are not the obstructive factors for the green transformation behavior of resource industries at this stage, and hypotheses H1a, H1b, H1f, and H3 are not confirmed.

Combining the above analyses and ranking the regression coefficients by the absolute values of the explanatory variables, it can be seen that poor talent, weak supply–demand linkage, high sunk cost, insufficient capital, and weak management innovation become the obstructive factors for the green transformation behavior of resource industries, and they are in decreasing order of constraint on the green transformation behavior of resource industries.

### 5.2. Expandability Analysis

Based on the original data and model (2), the provinces were grouped according to the northeastern, eastern, central, and western regions for studying the similarities and differences in the factors that impede the green transformation behavior of the resource industries in different regions. The results are displayed in Table 5.

The results in Table 5 demonstrate that the test results of the barriers to the green transformation behavior of the resource industries in Northeast China differ significantly from those of the nationwide test results. While the coefficients of the capital stock term and the sunk cost term are significantly negative at the 5% level, the coefficient of the market mechanism validity term is significantly positive at the 5% level, which indicates that the market mechanism validity is positively related to the green transformation efficiency of the resource industries. When the market mechanism validity is high, the market competition is intense and the marginal profit is lower. Providing stronger marginal and risk incentives will greatly promote the green transformation behavior of the resource industries. The weak market mechanism is also a barrier for the green transformation behavior of the resource-based enterprises in Northeast China. The coefficient of the mineral endowment term is significantly positive at the 5% level, which indicates that the mineral endowment is positively related to the green transformation efficiency of the resource industries. This implies that a considerable resource endowment can enhance the supply of the green transformation system. It can support the green transformation behavior, which indicates that the decrease in the mineral endowment will inhibit the green transformation behavior of the resource industries. Therefore, the gradual decline in the minerals is also a barrier to the green transformation behavior of the resource industries in Northeast China. The remaining barrier factor variables are not significant here. The fundamental reason for this conclusion is that the northeast region is an old industrial base with the tradition in deep planning management, weak market competition mechanism, poor business environment, insufficient foreign investment, and severe resource depletion. The barriers to the transformation behavior are more concentrated and highlighted.

The test results of the barriers to the green transformation behavior factors of the resource industries in the eastern region are similar to those of the nationwide test, ranked by the decreasing absolute value of the regression coefficients of explanatory variables, viz., poor talents, high sunk costs, and insufficient capital, except that the weak supply–demand linkage and weak management innovation are not significant. It indicates that the resource industries in the eastern region have developed earlier with newer development concepts, a wide range of green transformation behavior initiations, and high efficiency. The transformation behavior barrier factors are representative.

Compared with the whole country, the obstructive factors of green transformation behavior of resource industries in the central and western regions mainly focus on weak management innovation and a weak supply–demand linkage, while the test results of lack of talents, insufficient funds, and high sunk cost are not significant. It indicates that with the implementation of the Western Development Strategy and the Central Rising Strategy, the talent policy and capital policy are tilted to the central and western regions. The expected goal of conveying talents and supporting funds has been achieved. Meanwhile, the central and western regions have a vast territory, rich resources, late development of resource industries, low degree of transformation, and low sunk costs.

### 5.3. Robustness Tests

#### 5.3.1. Robustness Test I

To test the stability of the empirical results, considering that the environmental efficiency of the resource industries can respond to the green development status, the green transformation efficiency has been replaced by the environmental efficiency, keeping the explanatory variables and the applicable model unchanged, and the new explanatory variables were tested using Stata 16.0. The results are listed in Table 6.

According to Table 6, the coefficients of the talent level term and the management innovation ability term remain significantly positive at the 1% level, while the coefficients of the capital stock term, the sunk cost term, and the supply and demand correlation term remain significantly negative at the 1% and 5% levels, respectively, indicating that poor talent, weak management innovation, high sunk cost, insufficient capital, and weak supply–demand linkage can be obstructive factors for the green transformation behavior of resource-based enterprises. It can also be found that the coefficient of the technology reserve term becomes positive but still insignificant, which remains consistent with the previous results, while the barrier effects of mineral fading, poor location, and weak market mechanism remain unconfirmed, which is similar to the basic regression results, indicating that the previous regression results are basically robust.

#### 5.3.2. Robustness Test II

Considering that the Tobit model was adopted for the fundamental regression analysis, the model estimation will be biased once the perturbation terms have heteroskedasticity or defy the normality, since the Tobit model is nonlinear and cannot take methods such as the JB test. According to Wooldridge (2010) [52,53], if the Tobit model is set correctly, i.e., it satisfies normality and homoskedasticity, there is little difference between the estimated results of the Clad and Tobit models. The estimated results of the Clad model can be used to test the Tobit model. Therefore, by Stata 16.0, the regressions were performed on the original data based on the Clad model. The comparative results are shown in Table 7.

According to Table 7, the difference in the magnitude of the coefficient values of the variables calculated using the Clad and Tobit models is not significant. Since both are significant, the Tobit model can be considered to be correctly set, and this supports the robustness of the empirical results of the basic regression analysis.

## 6. Research Findings and Policy Implications

### 6.1. Research Findings

Based on the above systematic analysis of the GEM model and the results of the empirical test of the Tobit model, and further comparing the findings of Zhang et al. (2020) [54], Shao et al. (2018) [32], and Yuan et al. (2019) [55], the following conclusions can be drawn.

(1) Barriers to the green transformation behavior of the resource industries form a significant barrier effect through the inhibition of green transformation efficiency. Generally, the barrier effects of the five categories of factors, viz., “poor talent”, “insufficient capital”, “high sunk cost”, “weak supply–demand linkage”, and “weak management innovation”, are significant. The barrier effects of the four types of factors, viz., “poor location”, “weak technology reserve”, “weak market mechanism”, and “declining minerals”, are not significant.
(2) There is a difference in the intensity of the effects of the resource industries’ barriers to the green transformation behavior. Generally, there is a specific pattern in the intensity of the effect of the five types of barriers, viz., the highest intensity of the barrier effect of “poor talent” the intensity of the barrier effect of “weak supply and demand linkage”, “high sunk cost”“insufficient capital,” and “weak management innovation,” in descending order.
(3) A regional heterogeneity exists in the effects of the barriers to the green transformation behavior of the resource industries. In the northeast regions, the barrier effect of “weak market mechanism” is the strongest, and the barrier effects of “insufficient capital”, “high sunk cost”, and “declining minerals” are decreasing in order of intensity. In the eastern regions, the barrier effect of “poor talent” is the strongest, and the barrier effects of “high sunk cost” and “insufficient capital” are decreasing in order of intensity. In the central and western regions, the barrier effects of “weak management innovation” and “weak supply–demand linkage” are decreasing in order of intensity. The main reason for the differences in the barriers to the green transformation behavior of the resource industries in different regions, and the intensity of their effects, is the varying economic development levels and resource industries’ development stages pertaining to each region.

### 6.2. Policy Implications

The conclusions of this paper have important policy implications for the breaking down of the behavioral barriers to the green transformation of resource industries. This scientifically promotes the green, sustainable, and high-quality development of resource industries, accelerates the decarbonization process in emerging economies such as China and becomes the scientific basis for further improving the effectiveness of relevant policies. The policy implications and recommendations are as follows:

(1) Innovative green technology solves the technical bottleneck of the green transformation of resource industries. Emerging economies lag behind developed countries in green technology innovation capacity, and advanced low-carbon technologies are mainly dominated by developed countries. Hence, emerging economies should improve the whole life cycle of the green technology standard of the resource utilization and promote the efficiency of the green technology application. They also guide the resource industries to develop new resource exploitation and utilization technologies for the green mining, efficient recycling, clean conversion, and recycling from various links in the industrial chain. They can employ break through key technologies, build ecological, digital, and intelligent mines, and change the mode of production from the extensive to intensive, and from black to green, to minimize the consumption of the resources and environment, and maximize the social and economic benefits.
(2) Promote the management innovation and enhance the management capacity of resource industries for green transformation. In the age of information technology, economic globalization continues to develop deeply, and advanced management experiences are exchanged and spread among different emerging economies. The government should guide resource industries to use the mobile internet, big data, cloud computing, blockchain, artificial intelligence, and other information technologies to build a composite ecological management system. It facilitates the information management of the rate and intensity of resource development and the resourceful and harmless management of waste. Resource industries should implement the gradient green transformation and gradually share the high sunk costs. Concomitantly, they can carry out the material flow analysis, input–output analysis, and life-cycle evaluation to change the management decision from the linear thinking to systematic thinking and continuously improve the ability to engage in the ecological management and green transformation.
(3) Optimize the talent attraction and training to ensure the talent demand for the green transformation of resource industries. For emerging economies, the coordinated development of economy and ecology is inseparable from the transnational introduction and exchange of talents. Local governments should promote transnational exchange of talents for green development, based on the resource industry chain and international circulation. Additionally, they should also vigorously promote the market-oriented reform of factors and the free flow of the talent resources from universities and research institutes for green innovation practice activities, besides exploring the establishment of a“talent station”,“talent enclave”, and scientific research breeding base to realize the complementary advantages of the green development talents. The enterprises should vigorously implement the model of “talent + project + platform”, and give full play to the function and role of the platform to gather talents, a project to cultivate talents, and talents to drive the green transformation. Promoting enterprises, universities, and research institutes to form strategic alliances actively, and complementing each other’s strengths and weaknesses, are to jointly solve the critical problems in the green transformation development and create a long-term mechanism.
(4) Develop green finance to alleviate the lack of funds for the green transformation of resource industries. Emerging economies are in a period of rapid economic development, with capital concentrated on high profit projects and a lack of capital for green development. It is essential to implement differentiated financial policies and diversified financial products for different sunk cost enterprises, different transformation bases, and different transformation projects with different needs. The insurance industry lists a variety of green insurance to improve the innovation risks borne by the resource industries owing to green transformation according to the local conditions. Establishing a mechanism to sink the service center of gravity of the financial institutions is to grasp the dynamics and needs of the resource industries’ green transformation, promptly. Nevertheless, improving a highly adaptable green financial service system is to meet the capital needs of the enterprises’ green transformation.
(5) Optimize enterprise clusters and promoting the linkage between the supply and demand for the green transformation of resource industries. Enterprise clusters are the product of economic development to a certain stage, and emerging economies should learn from the experience of advanced resource industry cluster construction. Resource industries pay attention to the advantages of clusters, create interconnected green supply chains, and promote coupling among enterprises with close ecological relationships, including producers, suppliers, and customers. Each enterprise should promote the concentric diversification of the enterprises and explore the cross-industry diversification of the enterprises. Ecological chains should be built based on the by-product and waste exchange relationships among the enterprises, to strengthen the competitive relationships in the industrial chains among the homogeneous types of enterprises, and to promote symbiosis among the heterogeneous enterprises. The efficient circulation of the ecological media, such as the ecological concept and culture, by-products, and waste, can be accelerated to meet the needs of green transformation.

## 7. Limitations and Future Research

The phenomenon of the inconsistent pace of green transformation of the resource industries and transferring pollution by beggar-thy-neighbor reveals the existence of some factors that hinder the green transformation behavior of resource industries. This paper uses GEM model to systematically analyze the barrier factors of green transformation of the resource industries. Although the study comprehensively analyzed the barrier factors, it objectively lacked an in-depth study of a particular barrier factor, for example, the possible transformation from a barrier factor to a driver when certain variables reach a threshold. At the same time, in order to avoid the perturbation of input–output and barrier factors by unexpected events as well as to maintain the uniformity of the policy attitude of environmental protection, especially the larger impact of the sudden outbreak of the COVID-19 epidemic in 2020–2021, in order to discover the stable law in the process of green transition. Data from 2012–2019 were selected for this paper to examine the magnitude of the barriers, but to some extent weakened the timeliness of the study. Therefore, future studies are expected to further break through these two limitations and achieve greater results.

## Figures and Tables

**Table 2:** Setting and description of the barrier factor variables.

Barrier Factor Variables	Variable Representation	Measurements	Nature of Indicator	Data Source
Mineral endowment	res	Ratio of fixed assets of mining industry to fixed assets of the whole society by province	+	China Statistical Yearbook
Location status	tr	Annual tax revenue of mining industry by province	−	China Tax Yearbook
Talent level	edu	Average years of education	+	China Statistical Yearbook
Capital stock	alr	Ratio of liabilities to current assets in the mining industry by province	−	China Industrial Statistical Yearbook and Provincial Statistical Yearbooks
Sunk cost	dep	Accumulated depreciation of mining industry by province	+	Chinese Industrial Statistical Yearbook and Provincial Statistical Yearbooks
Technology reserve	cap	Green invention patent applications	+	CNRDS database
Supply and demand correlation	cn	Number of registered upstream and downstream employees in the mining industry by province	−	China Statistical Yearbook
Management Innovation capability	mc	Management innovation composite index	+	Chinese Industrial Statistical Yearbook and Provincial Statistical Yearbooks
Market mechanism validity	mi	Marketability index by province	+	China Marketization Index Report by Provinces

Note: Some of the data were obtained from the relevant yearbooks and further compiled and calculated by the authors.

**Table 3:** Descriptive statistics of the barrier factor variables.

Variable	Regional Group	Mean	Standard Deviation	Minimum	Maximum
res	Northeast region	0.1479	0.1210	0.0227	0.6886
	Eastern region	0.0331	0.0345	0.0000	0.1377
	Central and western Region	0.1701	0.1835	0.0705	1.0000
tr	Northeast region	0.2530	0.1546	0.0923	0.6199
	Eastern region	0.1701	0.1221	0.0000	0.5607
	Central and western region	0.2278	0.1875	0.0955	1.0000
edu	Northeast region	0.3936	0.0505	0.327	0.4957
	Eastern region	0.4135	0.2066	0.2053	1.0000
	Central and western region	0.2478	0.1011	0.0000	0.4499
alr	Northeast region	0.3419	0.1136	0.2139	0.7140
	Eastern region	0.2794	0.2119	0.0632	1.0000
	Central and western region	0.2147	0.1459	0.0000	0.7071
dep	Northeast region	0.4332	0.2423	0.0750	0.9825
	Eastern region	0.1782	0.2030	0.0000	0.8736
	Central and western region	0.2089	0.2425	0.0078	1.0000
cap	Northeast region	0.0527	0.0311	0.0109	0.1283
	Eastern region	0.2660	0.2638	0.0094	1.0000
	Central and western region	0.0681	0.0806	0.0000	0.4605
cn	Northeast region	0.3523	0.2350	0.1065	0.7516
	Eastern region	0.2444	0.2588	0.0000	1.0000
	Central and western region	0.4774	0.2482	0.0511	0.9758
mc	Northeast region	0.3008	0.1084	0.120	0.4903
	Eastern region	0.3388	0.1884	0.0000	0.6580
	Central and western region	0.3715	0.1912	0.062	1.0000
mi	Northeast region	0.4501	0.0397	0.3844	0.5175
	Eastern region	0.6794	0.1910	0.3021	1.0000
	Central and western region	0.3716	0.1675	0.0000	0.743

**Table 1:** Efficiency of the green transformation of resource industries in China, 2012–2019.

Province	Annual Green Transformation Efficiency							
	2012	2013	2014	2015	2016	2017	2018	2019
Beijing	1.00	1.06	1.25	1.91	4.04	2.62	2.11	2.67
Tianjin	0.97	1.00	1.00	0.95	1.29	1.21	1.16	1.17
Hebei	1.00	0.90	0.76	0.71	0.70	0.73	0.73	0.71
Inner Mongolia	1.00	1.00	0.94	0.86	1.00	1.00	0.72	0.75
Jiangsu	0.72	0.73	0.73	0.75	0.74	0.75	0.75	0.75
Zhejiang	1.00	1.00	1.01	0.76	0.83	0.73	0.76	1.00
Fujian	0.88	0.81	0.84	0.82	0.78	0.72	0.73	1.00
Shandong	1.00	1.00	0.99	0.87	0.83	0.93	0.88	0.70
Guangdong	0.87	1.00	0.71	0.72	0.72	0.73	0.73	0.71
Hainan	1.00	1.57	1.38	1.00	1.00	1.05	1.00	1.00
Liaoning	0.79	0.81	0.79	0.71	0.79	0.78	0.73	0.72
Jilin	0.78	0.80	0.80	0.82	0.83	0.87	1.20	0.83
Heilongjiang	0.76	0.76	0.76	0.81	0.84	0.77	0.76	0.77
Shanxi	1.00	0.95	0.95	0.93	0.95	0.97	1.00	1.00
Anhui	0.76	0.74	0.76	0.76	0.74	0.75	0.79	0.74
Jiangxi	0.79	0.72	0.78	0.70	0.69	0.72	0.76	0.73
Henan	1.00	0.95	0.96	0.89	0.84	0.76	0.73	0.73
Hubei	0.74	0.69	0.79	0.70	0.69	0.73	0.77	0.69
Hunan	1.00	1.00	0.78	0.69	0.71	0.75	0.81	0.69
Guangxi	0.89	1.00	0.84	0.84	1.00	0.73	0.96	0.71
Chongqing	0.79	0.76	0.80	0.83	0.88	1.01	0.99	0.83
Sichuan	0.85	0.71	0.71	0.72	0.72	0.72	0.73	0.72
Guizhou	0.72	0.70	0.71	0.73	0.73	0.75	0.79	0.73
Yunnan	0.72	0.70	0.73	0.74	0.72	0.80	0.78	0.71
Shanxi	0.96	1.01	0.96	0.86	0.88	0.90	1.00	1.00
Gansu	0.78	0.80	0.82	0.93	1.03	0.92	0.93	0.93
Qinghai	0.85	0.84	0.89	1.02	1.13	1.17	1.06	0.75
Ningxia	0.88	0.88	0.92	1.06	1.05	1.58	0.96	0.98
Xinjiang	0.74	0.73	0.74	0.77	0.80	0.77	0.74	0.74
National average	0.87	0.88	0.87	0.86	0.96	0.93	0.90	0.88
Eastern average	0.94	1.01	0.96	0.94	1.21	1.05	0.98	1.08
Northeast average	0.78	0.79	0.78	0.78	0.82	0.80	0.90	0.77
Central and western average	0.85	0.83	0.83	0.82	0.86	0.88	0.85	0.79

**Table 4:** Basic regression results.

Variables	Coefficient	Standard Deviation	T-Statistic	*p*-Value
res	−0.250 **	0.096	−2.600	0.010
tr	0.334 ***	0.084	3.960	0.000
edu	0.433 ***	0.076	5.730	0.000
alr	−0.175 ***	0.049	−3.540	0.000
der	−0.189 ***	0.048	−3.910	0.000
cap	−0.077	0.065	−1.170	0.243
cn	−0.217 ***	0.040	−5.410	0.000
mc	0.159 **	0.068	2.330	0.021
mi	−0.213 ***	0.076	−2.81	0.005
Constant term	0.907 ***	0.036	25.2	0.000

Note: *** and ** denote significant at the 1% and 5% levels, respectively.

**Table 5:** Regression results of the Tobit model for different regions.

Variables	Northeast Region	East Region	Central and Western Region
res	0.161 **	−1.926 **	−0.291 *
tr	0.035	1.071 ***	0.385 ***
edu	−0.746	0.838 ***	−0.187
alr	−0.566 **	−0.213 **	−0.105
dep	−0.221 **	−0.483 ***	−0.016
cap	−1.907 *	−0.090	−0.429 **
cn	−0.003	−0.156	−0.277 ***
mc	−0.456 **	0.202	0.298 ***
mi	1.644 **	−0.471 ***	−0.070
Constant term	0.841 ***	0.937 ***	0.946 ***

Note: ***, **, and * denote significant at the 1%, 5%, and 10% levels, respectively.

**Table 6:** Robustness regression results.

Variables	Coefficient	Standard Deviation	T-Statistic	*p*-Value
res	−0.634 ***	0.199	−3.180	0.002
tr	0.744 ***	0.175	4.260	0.000
edu	0.952 ***	0.137	6.960	0.000
alr	−0.358 ***	0.102	−3.500	0.001
dep	−0.523 ***	0.099	−5.270	0.000
cap	0.007	0.134	0.050	0.959
cn	−0.187 **	0.083	−2.270	0.024
mc	0.804 ***	0.142	5.670	0.000
mi	−0.381 **	0.152	−2.510	0.013
Constant term	0.289 ***	0.072	4.030	0.000

Note: *** and ** denote significant at the 1% and 5% levels, respectively.

**Table 7:** Comparison of Tobit and Clad models and the regression results.

Variables	Tobit Model	Clad Model
res	−0.250 *	−0.440 ***
tr	0.334 ***	0.191 ***
edu	0.433 ***	0.842 ***
alr	−0.175 ***	−0.158 ***
dep	−0.189 ***	−0.138 ***
cap	−0.0767	0.281 ***
cn	−0.217 ***	−0.224 ***
mc	0.159 *	0.224 ***
mi	−0.213 **	−0.753 ***
constant term	0.907 ***	1.007 ***
N	232	232

Note: ***, **, and * denote significant at 1%, 5%, and 10% levels, respectively.

## Data Availability

The data and materials are available from the corresponding author upon reasonable request.
